# Activation of Mitogen-Activated Protein Kinase in Descending Pain Modulatory System

**DOI:** 10.1155/2011/468061

**Published:** 2010-12-01

**Authors:** Hiroki Imbe, Emiko Senba, Akihisa Kimura, Tomohiro Donishi, Isao Yokoi, Yoshiki Kaneoke

**Affiliations:** ^1^Department of Physiology, Wakayama Medical University, Kimiidera 811-1, Wakayama City 641-8509, Japan; ^2^Department of Anatomy and Neurobiology, Wakayama Medical University, Kimiidera 811-1, Wakayama City 641-8509, Japan

## Abstract

The descending pain modulatory system is thought to undergo plastic changes following peripheral tissue injury and exerts bidirectional (facilitatory and inhibitory) influence on spinal nociceptive transmission. The mitogen-activated protein kinases (MAPKs) superfamily consists of four main members: the extracellular signal-regulated protein kinase1/2 (ERK1/2), the c-Jun N-terminal kinases (JNKs), the p38 MAPKs, and the ERK5. MAPKs not only regulate cell proliferation and survival but also play important roles in synaptic plasticity and memory formation. Recently, many studies have demonstrated that noxious stimuli activate MAPKs in several brain regions that are components of descending pain modulatory system. They are involved in pain perception and pain-related emotional responses. In addition, psychophysical stress also activates MAPKs in these brain structures. Greater appreciation of the convergence of mechanisms between noxious stimuli- and psychological stress-induced neuroplasticity is likely to lead to the identification of novel targets for a variety of pain syndromes.

## 1. Introduction

In human brain, there is a neural network that modulates the transmission of nociceptive messages, which is termed descending pain modulatory system. The cerebral cortex and amygdala project directly and indirectly via the hypothalamus to the periaqueductal grey (PAG). The PAG in turn controls spinal nociceptive neurons through relays in the rostral ventromedial medulla (RVM) and the dorsolateral pontine tegmentum (DLPT). The RVM consists of the nucleus raphe magnus (NRM), nucleus reticularis gigantocellularis pars alpha (GiA), and the ventral nucleus reticularis gigantocellularis (Gi) and is a major source of serotonergic projections to the spinal dorsal horn. The DLPT includes the noradrenergic neurons, such as the locus coeruleus (LC), A5 and A7, which are major sources of noradrenergic projections to the dorsal horn. These descending inputs especially from the RVM exert bidirectional (facilitatory and inhibitory) influence on nociceptive transmission in the spinal dorsal horn [1–3]. In earlier studies, attention has been mainly focused on the descending inhibitory influence. However, recently, it has also been known that the descending input from the RVM facilitates neuronal responses in the spinal dorsal horn and contributes to persistent pain and hyperalgesia [2–4]. Descending modulation is not a static process but exhibits dynamic changes in response to persistent noxious input following peripheral inflammation and nerve injury [5–7].

The mitogen-activated protein kinases (MAPKs) are a superfamily of intracellular signal transduction molecules that are evolutionally conserved [8, 9]. The MAPKs superfamily is made up of four main and distinct signaling pathways: the extracellular signal-regulated protein kinase1/2 (ERK1/2), the c-Jun N-terminal kinases or stress-activated protein kinases (JNK/SAPKs), the p38 MAPKs, and the ERK5. Each of MAPKs signaling pathways involves a consecutive activation of four levels of signaling molecules: small GTPases (Ras or Rac), MAPK kinase kinases (Raf or MAPKKKs), MAPK kinases (MEKs or MAPKKs), and MAPKs. The initial Ras and Rac localize to the inner surface of plasma membrane and transmit extracellular signals to downstream components of MAPKs cascades (MAPKKKs). MAPKKKs activate MAPKKs, which are dual-specific kinases that phosphorylate at both Ser/Thr and Tyr sites, targeting a Thr-X-Tyr motif on the MAPKs (where X is glutamate (ERK1/2, ERK5), proline (JNK), or glycine (p38 MAPK)). The MAPKs, serine/threonine kinases, are activated by MAPKKs. Phosphorylation of the MAPKs results in a conformational change and *a* > 1000-fold increase in specific activity [10–12]. At the end of these signaling pathways, active MAPKs phosphorylate their target molecules, many of which are transcription factors, leading to facilitation of target gene expression. Thus, it is well established that neural MAPKs cascades play important roles in synaptic plasticity and remodeling during induction of long-term potentiation (LTP), learning, and memory consolidation [13, 14].

 In the last 10 years, a number of studies have demonstrated that acute noxious stimuli, peripheral inflammation, and nerve injury activate MAPKs in several brain regions that are components of descending pain modulatory system [15–26]. These MAPKs activations play an important role in induction and maintenance of neural plasticity, which is thought to be essential for understanding the mechanism underlying dynamic changes in descending pain modulatory systems following peripheral tissue injury [5–7, 27]. To explore the role of each MAPK signaling pathway, the specific inhibitors such as ERK1/2 inhibitor (MEK inhibitor PD98059, U0126), p38 MAPK inhibitor (SB203580), and JNK inhibitor (SP600125) have been used in these studies [28]. The administrations of these inhibitors to descending pain modulatory systems alleviated hyperalgesia and allodynia in peripheral inflammatory pain models. [18, 21, 29]. In this paper, first, we introduce which MAPK is activated by such noxious stimuli and where those activations occur in descending pain modulatory system. Pain is a complex experience that involves not only the transduction of noxious environmental stimuli, but also cognitive and emotional processing in the brain [30]. Second, we discuss which of pain perception, pain-related emotional responses, and pain-related memory is the activation of MAPKs in these components related with it. 

Stress affects brain activity and promotes long-term changes in multiple neural systems. A variety of environmental and/or stressful stimuli have been shown to induce not only pain suppression but also an increase in pain sensitivity. These phenomena are termed stress-induced analgesia (SIA) and stress-induced hyperalgesia (SIH), respectively [31]. Stress has also been found to exacerbate and could contribute to the etiology of chronic painful disorders, such as, fibromyalgia [32], irritable bowel syndrome [33], rheumatoid arthritis [34], and headache [35]. Psychophysical stress also activates MAPKs in brain structures related to descending pain modulatory system. MAPKs-induced neural plasticity in some of these structures might be associated with “limbically augmented pain syndrome” [36]. In this theory, stress and emotionally traumatic events lead to a sensitization of corticolimbic structures, which subserve both nociceptive processing and affective regulation. Therefore, we also discuss stress-induced activations of MAPKs in these structures.

## 2. Activation of Mitogen-Activated Protein Kinase in Descending Pain Modulatory System

### 2.1. Rostral Ventromedial Medulla (RVM)

**Figure 1:**
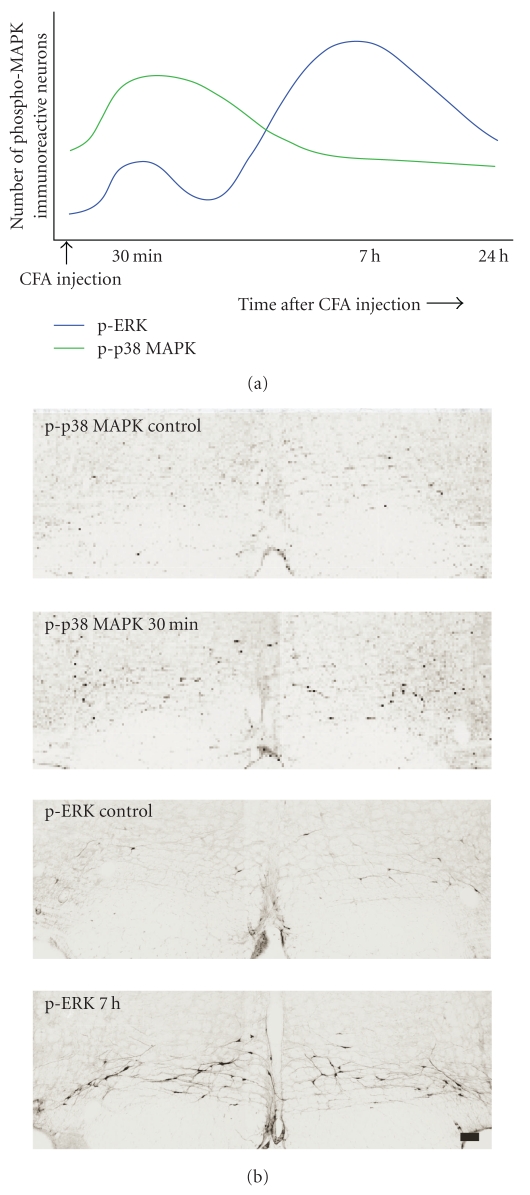
(a) Time courses of p-ERK1/2 and p-p38 MAPK in the RVM after CFA injection into the hindpaw. (b) Photomicrographs showing p-ERK1/2- and p-p38 MAPK-immunoreactive neurons in the RVM (bregma −11.00 mm) following hindpaw inflammation. Scale  bar = 100 *μ*m.

Peripheral inflammation induced by CFA injection into the hindpaw activated ERK1/2 and p38 MAPK in the RVM. The activation of ERK1/2 exhibited two characteristic phases. The first phase was a transient small increase at 30 minutes after CFA injection. The second phase was more persistent and pronounced increase from 3 hours to 24 hours, with a peak at 7 hours [15]. On the other hand, the activation of p38 MAPK was more short lived. It peaked at 30 minutes and lasted for 1 hour [16] (Figure 1). Phosphorylated ERK1/2 and p38 MAPK (p-ERK1/2 and p-p38 MAPK) were present predominantly in RVM neurons after CFA injection. About 60% of p-ERK1/2 neurons and 40% of p-p38 MAPK neurons in the RVM were serotonergic neurons [15, 16]. Microglial p-p38 MAPK in the RVM has also been reported to increase following carrageenan-induced inflammation [18].

Microinjection of U0126, an MEK inhibitor, into the RVM partially restored a decrease of paw withdrawal latency to noxious heat stimulus into the inflamed hindpaw [29]. ERK1/2 is involved in both transcription-independent and transcription-dependent forms of central sensitization. The former is early onset process, such as phosphorylation of receptors and trafficking of receptors to the synapse, and the latter is late onset, such as an increase in the expression of late-response genes [27, 37]. Since the microinjection of U0126 into the RVM significantly attenuated thermal hyperalgesia at 24 hours, but not at 7 hours after CFA injection [29], activation of ERK1/2 in the RVM might be involved in transcription-dependent plasticity. It has been demonstrated that the phosphorylation of ERK1/2 activates the transcription of tryptophan hydroxylase (TPH), the rate-limiting enzyme in serotonin biosynthesis, in the serotonergic neuron-like cell line [38]. Thus, activation of ERK1/2 in RVM serotonergic neurons is assumed to increase serotonin biosynthesis. 5-hydroxytryptamine (5HT) released from the descending bulbospinal neurons seems to exert dual (inhibitory and facilitatory) effects on spinal nociceptive processing [2, 3]. Oyama et al. [39] reported that the inhibitory and facilitatory effects were mediated by 5HT1A and 5HT3 receptors, respectively. Nearly one-half of DRG neurons projecting to the superficial dorsal horn express 5HT3 receptor [40], and activation of 5HT3 receptor localized on central terminals of DRG neurons seems to enhance the release of neuropeptides [41]. Recently, by depleting endogenous 5HT in the RVM serotonergic neurons, it has been demonstrated that the RVM 5HT system participates in descending pain facilitation but not descending inhibition, which is necessary for maintenance of hyperalgesia and allodynia after peripheral inflammation and nerve injury [42]. Thus, it is possible that ERK activation induced by inflammation increases the transcription of TPH and serotonin biosynthesis, leading to the enhancement of hyperalgesia via descending serotonergic pathways.

 Microinjection of SB203580, a p38 MAPK inhibitor, into the RVM attenuated carrageenan-induced thermal hyperalgesia and tactile allodynia [18]. The activation of p38 MAPK is involved in tumor necrosis factor-*α* and interleukin-1 production [43]. Since these cytokines phosphorylate NMDA receptor in the RVM neurons and cause allodynia [44], glial p38 MAPK may contribute to descending pain facilitation via cytokine production. In CA1 pyramidal neurons, a small amount of Ca^2+^ ion influx via NMDA receptor activates the Rap-p38 MAPK signaling pathway, which drives the removal of synaptic AMPA receptors [45]. The activation of neuronal p38 MAPK in the RVM may contribute to a decrease in RVM excitability via the removal of synaptic AMPA receptors. AMPA receptor sensitivity in the RVM was reduced at 3 hours after the hindpaw inflammation, [46, 47]. Phosphorylation of p38 MAPK has also been shown to activate the transcription of TPH [38]. The activation of p38 MAPK in RVM serotonergic neurons may also contribute to serotonin biosynthesis. Thus, it is possible that p38 MAPK activation induced by inflammation increases glial cytokines and neuronal TPH production, leading to the enhancement of hyperalgesia and allodynia.

 Chronic restraint stress (6 h daily for 3 weeks) induced thermal hyperalgesia and significant increase in activation of ERK1/2 in the RVM [48]. This stress-induced ERK1/2 activation in the RVM serotonergic neurons may also contribute to serotonin biosynthesis. The level of TPH in the RVM was significantly increased in the rats with chronic restraint stress [48]. Meanwhile, many studies have reported that chronic stresses decrease growth-associated and cytoskeletal proteins and induce neuronal atrophy in the hippocampus [49–51]. Since the sustained activation of ERK1/2 has been shown to be involved in neuronal degeneration [52], the stress-induced activation of ERK1/2 may be associated with neuronal atrophy and dendritic reorganization in the RVM.

### 2.2. Locus Coeruleus (LC)

Acute noxious stimulation induced by formalin injection into the hindpaw activated ERK1/2 in the LC for 1 hour after the injection. However, CFA-evoked chronic inflammation did not induce a prolonged activation of ERK1/2 in the LC. After formalin injection, p-ERK1/2 was almost exclusively (more than 90%) located in the tyrosine hydroxylase- (TH-) positive neurons of the LC [17].

 TH is the rate-limiting enzyme in NA biosynthesis. Short-term regulation of TH is accomplished by changes in the phosphorylation of this enzyme. ERK1/2 phosphorylates Ser_31_ in TH [53, 54]. The phosphorylation of Ser_31_ potentiates TH activity [55]. The activation of ERK1/2 in the LC after formalin injection might contribute to phosphorylation of TH and potentiation of TH activity. Furthermore, it has been shown that p-ERK1/2 activates c-fos, Fra-2, and CREB [56, 57]. The first two and the last interact with the AP-1 and CRE sites of the TH gene promoter, respectively [58]. Therefore, it has been speculated that activation of ERK1/2 in the LC increases TH gene transcription through the activation of several transcription factors [59, 60]. The activation of ERK1/2 in the LC might increase TH gene transcription and NA biosynthesis.

 Several studies have demonstrated that acute restraint stress increases p-ERK1/2 in the LC [17, 59–61]. On the other hand, chronic restraint stress (repeat 2–6 times) induced further marked activation of ERK1/2, JNK, and p38 MAPK in the LC [59]. Other study has reported that chronic restraint stress for 3 weeks decreases p-ERK1/2 in the LC [48]. The disparity among those experimental results may be due to the differences in duration of restraint stress and experimental protocol. Short-term anaesthesia has been shown to induce ERK1/2 phosphorylation in the brainstem [62]. LC neurons in waking animals are very responsive to nonnoxious auditory, visual, and somatosensory stimuli in the environment [63–66]. Thus, we must take careful note of anesthesia, handling of animals, and experimental environment to evaluate an activation of MAPK in the LC.

### 2.3. Periaqueductal Grey (PAG)

There are few studies that investigate activation of MAPK in the PAG. Some studies have reported the activation of ERK1/2 in the PAG after visceral noxious stimulation [19, 20]. Intraperitoneal injection of acetic acid significantly activated ERK1/2 in the PAG [19, 20]. The PAG has a longitudinal columnar organization, and each PAG column coordinates a distinct pattern of behavioral and physiological reactions critical for survival. The lateral and ventrolateral cell columns contain many neurons that project to the RVM [67]. Many p-ERK1/2 neurons were found in the lateral, ventrolateral, and dorsal columns. However, the densities of p-ERK1/2 neurons in these columns were not significantly different [20]. These results indicate that ERK1/2 is activated in several PAG neurons related to the different functional activity such as fear, anxiety, defensive reactions, and autonomic regulation in response to nociceptive stimuli.

### 2.4. Amygdala

The amygdala is now recognized as an important player in the emotional-affective dimension of pain [68, 69]. It has also been demonstrated that this structure modulates nociceptive behavior by affecting the activity of RVM [70, 71]. Peripheral inflammation activated ERK1/2 in the amygdala at 3 h after formalin injection into the hindpaw. Formalin-induced p-ERK1/2 neurons were almost exclusively located in the laterocapsular division of the central nucleus of the amygdala (CeLC) [21]. It is noteworthy that ERK1/2 activation was seen in the right CeLC, independent of the side of peripheral inflammation. Inhibition of ERK1/2 activation in the amygdala by U0126 significantly decreased mechanical but not thermal hypersensitivity. Pharmacological activation of ERK1/2 in the amygdala induced mechanical hypersensitivity in the absence of inflammation. These results have clearly shown that ERK1/2 activation in the amygdala plays a pivotal role in inflammation-induced mechanical hypersensitivity. ERK1/2 activation mediates plasticity in various brain regions. ERK1/2 activation in the CeLC neuron also contributes to synaptic facilitation by increasing NMDA receptor function after peripheral inflammation [72]. Furthermore, it has been demonstrated that ERK1/2 activation in the CeLC is downstream of metabotropic glutamate receptor 5 (mGluR5) [73]. The level of mGluR5 in the right amygdala was higher than that in the left amygdala. This seems to be one of the mechanisms for hemispheric lateralization of pain processing in the amygdala. 

 About the stress-induced activation of MAPKs, forced swim stress activated JNK, but not ERK1/2 and p38 MAPK, in the amygdala [74, 75]. The JNK signaling pathway is a major regulator for activation and expression of the AP-1 transcriptional factors such as c-Jun, c-Fos, and ATF. The activation of JNK may participate in stress-induced plastic change of the amygdaloid neurons. Maternal deprivation in early life increased immobility time in forced swim test and activation of ERK1/2 in the amygdala. Microinjection of PD98059 into the amygdala suppressed the immobility time. Thus, ERK1/2 activation in the amygdala seems to be implicated in the formation of depressive-like behavior [76].

### 2.5. Cerebral Cortex

The cerebral cortices including the anterior cingulate (ACC) and prefrontal cortices (PFCs) are believed to play important roles in emotion, learning, memory, and persistent pain in the adult brain [77–79]. The electrical stimulation of the ACC produces facilitation of tail-flick reflex induced by noxious heating via the RVM [80]. Formalin injection into the hindpaw activated ERK1/2 in rostral ACC (rACC) bilaterally [22]. A significant increase of ERK1/2 activation in the rACC occurred at 3 min, peaked at 10–30 min, and declined at 2 h but still remained after 24 h. Inhibition of ERK1/2 activation in the rACC by microinjection of PD98059 did not affect formalin-induced nociceptive behavior. However, PD98059 inhibited acquisition of formalin-induced conditioned place avoidance (F-CPA), which is believed to reflect pain-related negative emotion. ERK1/2 activation in the rACC required NMDA receptor, and it mediated CREB phosphorylation. However, the target genes regulated by CREB in the rACC remain to be elucidated. The activation of MAPKs including ERK1/2, JNK, and p38 MAPK has been shown to be critical for induction of LTP in the ACC [81]. The results have demonstrated that ERK1/2 activation in the rACC is critical for the development of affective pain (pain-related emotional response) but not nociceptive pain [22]. Peripheral nerve injury, such as digit amputation, also activated ERK1/2 in the ACC at 2 weeks after amputation [23]. Nonnoxious mechanical stimulation by brushing the hindpaw with amputated digit increased further the number of p-ERK1/2 neurons in the ACC and p-ERK1/2 in the dendrite and synaptic sites. The activation of ERK1/2 at the synaptic sites is thought to be involved in rapid synaptic potentiation and regulation of neuronal excitability [81, 82]. 

 Acute stress such as restraint and forced swim activates ERK1/2 and JNK in the PFC and cingulate cortex [74, 83]. On the other hand, the effects of chronic stress on MAPKs activation in the PFC are inconsistent across the previous studies. Chronic restraint and forced swim stresses decreased ERK1/2, JNK, and p38 MAPK activations in the PFC [83–85]. In contrast, chronic stress induced by inescapable footshock increased ERK1/2 activation in the PFC [86, 87]. What is the functional role of stress-induced alteration of MAPK activity in the PFC? Single-prolonged stress (SPS) consists of restrain for 2 h, forced swim for 20 min and ether anesthesia, and exposure to SPS activated ERK1/2 in the mPFC. Inhibition of ERK1/2 activation in the mPFC ameliorated stress-induced anxiety-like behavior, learning, and spatial memory impairment [88]. Antidepressant reversed stress-induced reduction of ERK1/2 activation in the PFC [85]. ERK1/2 activity in the PFC may be critical to depressive-like behavior and memory impairment. The rats subjected to prenatal stress showed a decrease of p38 MAPK activation in the PFC [89]. In these stressed rats, protein phosphatase-2A that dephosphorylates all MAPKs has been found to increase in the PFC. It has also been reported that p38 MAPK activation is involved in LTD at excitatory synapses of PFC pyramidal neurons [90]. These stress-induced reductions of MAPKs activation may impair synaptic plasticity. On the other hand, since sustained activation of MAPK induces neuronal degeneration [52], it is speculated that chronic stress-induced ERK1/2 activation may cause neuronal atrophy and reorganization [86, 87].

### 2.6. Hypothalamus

Electrical stimulation or opioid microinjection in the hypothalamus produces analgesia, which has been considered to play an important role in the modulation of pain. Beta-endorphin neurons in the hypothalamic arcuate nucleus (Arc) project to the PAG and activate descending projection neurons to the RVM in the PAG by inhibiting inhibitory GABA-ergic interneurons. This neural circuit has been implicated in the production of stimulation-produced and stress-induced analgesia [1, 2, 91]. Formalin injection into the hindpaw activated ERK1/2 in the hypothalamus [24]. ERK1/2 activation markedly increased at 30 min and remained higher than baseline after 24 h. p-ERK1/2 was colocalized with beta-endorphin in the Arc neurons. Proopiomelanocortin (POMC) is a precursor to several active peptides, including beta-endorphin. The i.c.v. injection of PD98059 attenuated formalin-induced increase of POMC mRNA expression in the hypothalamus. These results indicate that ERK1/2 activation in the hypothalamus may contribute to neuroendocrine regulation [24]. ERK1/2 activation in the hypothalamic paraventricular nucleus (PVN) has also been reported after intraplantar formalin or i.t. SP injections [25, 26]. The i.c.v. injection of PD98059 attenuated the second phase of formalin-induced nociceptive behavior [26]. Therefore, ERK1/2 activation in the PVN may be involved in acute nociceptive behavior.

 Acute restraint stress activated ERK1/2 in the Arc and the PVN [60, 92]. Acute swim stress also activates JNK in the hypothalamus [75]. However, chronic restraint stress did not activate ERK1/2 in the Arc and the PVN [60]. These activations might be involved in autonomic and endocrine responses to the stress. Those functions remain elusive, however.

## 3. Conclusions and Perspectives

**Figure 2:**
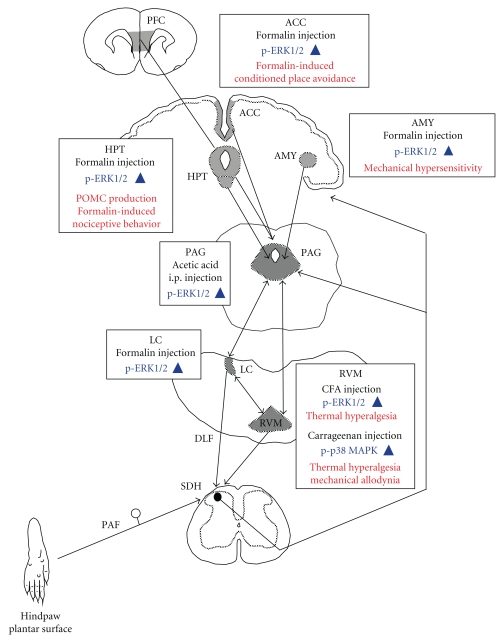
Schematic drawing of noxious stimuli-induced MAPKs activations in the descending pain modulatory system. Boxes indicate noxious stimulation, activated MAPK, and function that is related to MAPK activation. PFC, prefrontal cortex; ACC, anterior cingulate cortex; AMY, amygdala; HPT, hypothalamus; PAG, periaqueductal gray; LC, locus coeruleus; RVM, rostral ventromedial medulla; DLF, dorsolateral funiculus; SDH, spinal dorsal horn; PAF, primary afferent fiber; POMC, proopiomelanocortin. Upward and downward arrowheads indicate increase and decrease, respectively.

**Figure 3:**
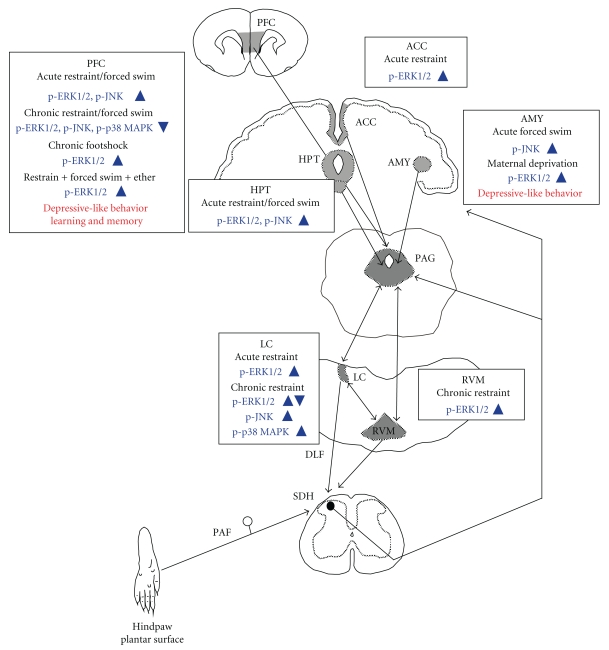
Schematic drawing of stress-induced MAPKs activations in the descending pain modulatory system. Boxes indicate psychophysical stress, activated MAPK, and function that is related to MAPK activation. For abbreviations see Figure 2.

The noxious stimuli induced activations of MAPKs in the components of descending pain modulatory system. The activations in these components were associated with pain perception and pain-related emotional responses (Figure 2). In addition, psychophysical stress also activated MAPKs in these structures. They seem to be mainly related to depressive-like behavior (Figure 3). MAPKs are involved in both transcription-independent and transcription-dependent forms of central sensitization. Stress-induced neural plasticity in these structures via activations of MAPKs might affect nociceptive processing. In turn, the noxious stimuli-induced neural plasticity might potentiate depressive-like behavior in response to psychological stress. Clinical studies have demonstrated a reciprocal interaction between emotionality and pain perception in chronic pain conditions [93]. Elucidation of their physiological functions might contribute to a better understanding of chronic pain and lead to the development of new treatment to a variety of pain syndromes.

 Recently, several studies have demonstrated that activation of ERK5 in the dorsal root ganglion and the spinal dorsal horn is related to hyperalgesia and allodynia following peripheral tissue injury [94–96]. Although there is no study that has examined the noxious stimuli-induced ERK5 activation in the supraspinal structures such as descending pain modulation system, one study has reported a reduction of ERK5 activity in the frontal cortex following psychophysical stress [97]. More importantly, PD98059 and U0126, specific inhibitors of MEK1/2-ERK1/2, also inhibit MEK5-ERK5 pathway [98]. Therefore, further studies are needed to examine whether the noxious stimuli induce ERK5 activation in descending pain modulation system and, if so, to explore what kinds of functions that activation is related to.
